# In Vitro and In Vivo Evaluation of a Bio-Inspired Adhesive for Bone Fixation

**DOI:** 10.3390/pharmaceutics15041233

**Published:** 2023-04-13

**Authors:** Matthias Schlund, Julien Dartus, Sarah Defrançois, Joël Ferri, Jérôme Delattre, Nicolas Blanchemain, Patrice Woisel, Joël Lyskawa, Feng Chai

**Affiliations:** 1 Univ. Lille, Inserm, CHU Lille, U1008—Controlled Drug Delivery Systems and Biomaterials, 59000 Lille, France; 2 Univ. Lille, Inserm, CHU Lille, Service de Chirurgie Maxillo-Faciale et Stomatologie, 59000 Lille, France; 3 Univ. Bordeaux, CHU Bordeaux, Service de Chirurgie Maxillo-Faciale et Stomatologie, 33000 Bordeaux, France; 4 Univ. Lille, UMET, CNRS, INRAE, Centrale Lille, UMR 8207—UMET, 59000 Lille, France; 5 Univ. Lille, Univ. Littoral Côte d’Opale, CHU Lille, ULP 4490—MABLab—Adiposité Médullaire er Os, 59000 Lille, France

**Keywords:** bone adhesive, bioresorbable adhesive, animal model, bone fixation, bone glue

## Abstract

Compared to metallic hardware, an effective bone adhesive can revolutionize the treatment of clinically challenging situations such as comminuted, articular, and pediatric fractures. The present study aims to develop such a bio-inspired bone adhesive, based upon a modified mineral-organic adhesive with tetracalcium phosphate (TTCP) and phosphoserine (OPS) by incorporating nanoparticles of polydopamine (nPDA). The optimal formulation, which was screened using in vitro instrumental tensile adhesion tests, was found to be 50%_mol_TTCP/50%_mol_OPS-2%_wt_nPDA with a liquid-to-powder ratio of 0.21 mL/g. This adhesive has a substantially stronger adhesive strength (1.0–1.6 MPa) to bovine cortical bone than the adhesive without nPDA (0.5–0.6 MPa). To simulate a clinical scenario of autograft fixation under low mechanical load, we presented the first in vivo model: a rat fibula glued to the tibia, on which the TTCP/OPS-nPDA adhesive (n = 7) was shown to be effective in stabilizing the graft without displacement (a clinical success rate of 86% and 71% at 5 and 12 weeks, respectively) compared to a sham control (0%). Significant coverage of newly formed bone was particularly observed on the surface of the adhesive, thanks to the osteoinductive property of nPDA. To conclude, the TTCP/OPS-nPDA adhesive fulfilled many clinical requirements for the bone fixation, and potentially could be functionalized via nPDA to offer more biological activities, e.g., anti-infection after antibiotic loading.

## 1. Introduction

As a global public health issue [1], bone fractures are commonly treated by immobilization to restore the normal alignment and anatomy of the bone, which often involves surgical techniques using metallic hardware (plates, screws, and pins). Unfortunately, these metallic hardware items poorly adapt to certain specific situations, such as comminuted [2] or intra-articular fractures [3], and pediatric fractures in a growing skeleton [4]. Moreover, there may be mechanical or infectious complications associated with such devices, which require surgical removal for resolution [5]. However, hardware removal surgery may cause postoperative morbidity [6], which meanwhile generates a heavy economic burden [7].

A bioresorbable and biodegradable bone glue would therefore be an excellent alternative, which will be much easier and safer to use in patients with comminuted or intra-articular fractures, and will be better adapted to the pediatric skeletal growth, thereby avoiding hardware-related complications and removal procedures. Furthermore, its use would not only be limited to traumatic bone fracture, but could also be extended to any surgery requiring bone healing or fusion such as osteotomies, bone graft fixation, and spinal fusion. It may also be used in conjunction with metallic implants, such as dental or arthroplastic implants, to enhance their primary stability. Despite such an evident need, there is currently no bone adhesive in clinical use that offers a strong enough bond and safe healing for wet bone.

The review by Farrar in 2012 [8] listed a range of properties that an ideal bone adhesive should possess. The necessity to bond and set in a wet environment thus remains the main challenge for the creation of a novel bone adhesive in comparison with common adhesives. Several synthetic adhesives have been elaborated based on materials such as polycyanoacrylates, poly(methyl methacrylate) (PMMA), polyurethane, and thiol-ene composites [9]. However, these non-resorbable synthetic materials are plagued by tissue toxicity, thus limiting their uses as bone adhesives. Currently, there is no biocompatible and bioresorbable bone adhesive available for a strong enough bond to wet bone that meets the clinical requirements. 

In this context, a biomimetic approach would be the best solution to toxicity issues. Calcium phosphate cements (CPCs) are the most widely used bone substitute material in the field of bone regeneration [10] due to their chemical similarity to the inorganic phase of bone (hydroxyapatite) and their excellent biological properties [11,12]. The properties of different CPCs can vary with their Ca/P ratio. Thanks to their greater solubility under neutral pH conditions compared to other CPCs, tetracalcium phosphate (TTCP) and α-tricalcium phosphate (α-TCP) are known for forming a paste with water, which progressively sets and hardens into a cement through a dissolution/precipitation process. Unfortunately, these cements have no intrinsic adhesive property to bone [13], thus limiting their use as bone adhesives.

Recently, inspired by marine creatures, notably the sandcastle worm (*Phragmatopoma californica*) [14], CPC-based bone adhesives were elaborated [15,16,17] by the addition of a phosphorylated amino acid, such as phosphoserine (OPS). Indeed, this key component of the sandcastle worm glue could self-reticulate with certain CPCs and form a biocompatible organo-mineral adhesive [15,16,17]. Among them, Tetranite^®^ adhesive (Revbio, Lowell, Massachusetts, United States), which is composed of TTCP and OPS, self-sets to a multiphasic solid within a few minutes. It was claimed to display high bone-to-bone and bone-to-metal adhesive strength in a wet environment, in addition to excellent bioresorbability, osteoconductivity, and biocompatibility [15,18,19,20]. Pujari-Palmer et al. [16,21,22,23,24] have also reported a bone adhesive (OsStic^TM^), based on OPS, α-TCP, and calcium metasilicate, with a similar performance as Tetranite^®^. Therefore, the association of OPS and CPC seems to be a promising direction to develop high bond strength bone adhesives. These CPC/OPS bone adhesive materials are hybrid materials that have some similar limitations to CPCs, notably their mechanical properties and specifically their low adhesion, low strength, and brittleness, that often restrict their wider use and confine them to mostly non-loadbearing applications. Subsequently, considerable effort is needed to improve their mechanical properties.

A bio-inspired strategy may be applied to CPC/OPS bone adhesives to further improve their mechanical and/or biological properties. Indeed, polydopamine (PDA), which has a similar structural to key components of mussel foot adhesive proteins [25], has sparked considerable interest as a biomimetic and versatile coating for a wide range of materials including biomaterials [26,27]. In particular, thanks to its latent reactivity towards nucleophiles, PDA was used as a universal platform for surface biofunctionalization to promote the osteointegration of bone substitutes [28]. In the presence of amino acid-like lysine or OPS, PDA is able to anchor to various wet surfaces [29]. When combined with a CPC cement, PDA was shown to boost osteogenesis and osteointegration [30,31,32]. Nanoparticles of polydopamine (nPDA), which are formed by the self-polymerization of dopamine in basic conditions, exhibited remarkable drug loading capacity [33] in addition to all properties of PDA [34,35,36]. Therefore, nPDA could be an ideal candidate for further improving the overall performance of CPC/OPS bone adhesive.

As well as the major need for the development of adequate bone adhesives, the methods of evaluation and further translation of adhesive candidates into clinical use also face a challenge. Several mechanical testing methods for evaluating the adhesive strength and the compressive resistance of bone adhesives in vitro/ex vivo have been described [15,16,18,19,37,38,39,40], showing a wide range of bone bond strength values depending on the type of bone along with sample surface preparation and testing conditions. So far, biomimetic CPC/OPS glues have been evaluated in vivo mostly regarding their biocompatibility, osseointegration, or bioresorbability [15,18,20,23,24]. However, animal models used for assessing their in vivo efficacy are scarcely reported [41]. Several applications for bone fixation were performed, but only in the regions without mechanical stress, such as the calvaria [20], or together with screws [18], which does not fully demonstrate the adhesive property of the bone glue. Therefore, a novel animal model, which demonstrates the in vivo efficacy of a bone adhesive over a clinically relevant time frame, is highly demanded. 

Thus, the present study aims to develop a bio-inspired bone adhesive based on a combination of TTCP, OPS, and nPDA. The formulation optimization and a thorough evaluation of mechanical properties and biomineralization of this adhesive were firstly performed. Then, a novel rat model of bone autograft fixation was designed and used to evaluate the in vivo biocompatibility and the adhesive property of the optimized glue. 

## 2. Materials and Methods

### 2.1. Materials

TTCP powder was purchased from Matexcel^®^ (Bohemia, NY, USA). OPS powder was purchased from Merck^®^ (Darmstadt, Germany). nPDA was synthetized according to the protocol of Ju et al. [42]. Briefly, 900 mg of dopamine hydrochloride was dissolved in 450 mL of pure water and heated to 50 °C, and then 3.8 mL of sodium hydroxide was added and stirred at 50 °C for 5 h before being purified by dialysis against deionized water (1 kD, Spectra/Por^®^ 7, Repligen, MA, USA) for 48 h. The dialysate containing a suspension of nPDA was finally freeze-dried to obtain nPDA powder. The characterization of the nPDA was conducted using dynamic light scattering analysis to determine the size distribution profile of the nPDA particles in suspension and with scanning electron microscopy (SEM) to observe the morphology of the nPDA.

An in vitro/ex vivo mechanical evaluation of the glue adhesion was made with three types of materials depending on the specific objective. Because most fixation hardware is made of titanium and because it is easier to obtain a sufficient number of standard samples from titanium than from animal bone, it is more practical to use titanium on a large scale for a screening test. Titanium cylinders (16 mm in diameter and 20 mm in height), cut from titanium bars (commercially pure titanium grade 4, Goodfellow^®^, Huntingdon, United Kingdom), were thus used for the formulation optimization of the glue. 

It was later necessary to assess the adhesive property of the optimized glue to ex vivo bone tissue: bovine cortical bone and rat tibia/fibula. Briefly, cow femurs were obtained from a local butcher’s shop, and were cut into rectangular cuboid samples with a bonding surface between 100 and 180 mm^2^ and stored at −20 °C before the test. The bonding surfaces of the bovine bone and titanium cylinders were polished with abrasive paper (P1200). The rat tibia and fibula were freshly harvested before the test by “recycling” rat (Sprague Dawley) cadavers from another research project to limit the number of animals used (‘3R’ principles). The external surface of the harvested tibia was firstly ground slightly with a dental bur, and the fibula segment (6 mm long, bonding surface of approx. 6 mm^2^) was then glued to the tibia for the tensile adhesion test, which is described later in Section 2.4.2.

### 2.2. Glue Fabrication

TTCP and OPS powders w/o nPDA were weighed precisely, according to the defined molar percentage (%_mol_) of OPS in the TTCP/OPS mixture and weight percentage (%_wt_) of nPDA, premixed with a spatula, and then ground with a pestle and mortar to obtain a homogeneous mixture. Though TTCP/OPS can form the glue equally as well with water, phosphate-buffered saline (PBS, pH 7.4) was selected (to limit pH variation) and added with a micropipette into powder, at predetermined liquid-to-powder ratios. All were mixed with a spatula for 20 s to obtain a smooth sticky paste, which was then rapidly applied onto the surface for gluing.

### 2.3. Impact of the Formulation on the Adhesion Force of the Glue

#### 2.3.1. Tested Glue Formulations

The effect of various formulation parameters on the adhesive strength of the glue was assessed using an instrumental tensile adhesion test (Section 2.3.2) on glued titanium cylinders. The molar percentage of OPS (30%_mol_, 45%_mol_, 50%_mol_, 55%_mol_, and 65%_mol_) in the TTCP/OPS mixture, the liquid-to-powder ratio (0.17, 0.21, and 0.25 mL/g), and the weight percentage of the nPDA (2%_wt_, 5%_wt_, and 10%_wt_) were screened in order to obtain the optimal formulation (with the highest adhesive strength).

#### 2.3.2. Instrumental Tensile Adhesion Test on Glued Titanium Samples

A thin layer of the glue, fabricated as described in Section 2.2, was applied on the polished surfaces of paired titanium cylinders, which were then glued together in a butt-joint configuration. The glued cylinder pair was manually compressed for 4 min and then immersed in a 37 °C PBS bath, simulating the aqueous physiological environment, for 1 or 24 h.

The adhesion strength was evaluated with mechanical traction until rupture (minimum N = 8 for each experiment) on an Instron 4466^®^ device (Norwood, Massachusetts, United States) with a 10 kN load cell at a displacement speed of 0.1 mm/s. The results were described as maximal traction stress (i.e., the force of failure per surface unit) applied on the glued samples.

### 2.4. Characterization of the Optimized Glue

#### 2.4.1. Ex Vivo Instrumental Tensile Adhesion Test on Glued Bovine Bone

An ex vivo instrumental tensile adhesion test was performed with the Instron 4466^®^ device, similarly to that stated above (Section 2.3.2) except for using a load cell of 1 kN on glued bovine cortical bone cuboid samples (minimum N = 8 for each experiment). The results were described as the maximal traction stress (the force of failure per surface unit) applied to the glued samples.

#### 2.4.2. Ex Vivo Manual Tensile Adhesion Test on Glued Rat Tibia/Fibula

An ex vivo manual tensile adhesion test was designed and performed on the glued rat tibia/fibula due to the non-conformed sample dimensions of the Instron device. Compared to the laboratory experimental conditions in the abovementioned instrumental test mode, the following described manual tensile adhesion test mode better mimics a clinical scenario of autografts fixation with the glue. A thin layer of the prepared glue was first applied to the ground lateral surface on the shaft of the tibia. A 4/0 Vicryl^®^ thread (Ethicon, Raritan, NJ, USA) was deposed in the middle of the applied glue, perpendicular to the long axis of the tibia, and then the fibula bar sample (autograft) was glued to the tibia under manual compression for 4 min. The glued samples were then immersed in a 37 °C PBS bath for 1 or 24 h. An evaluation of the adhesive strength was performed by traction with standard weights attached to the Vicryl^®^ thread (minimum N = 7 for each experiment). As shown below, by progressively increasing the added weights until rupture of adhesion, the sum of the applied weights was noted as the traction force of failure. The results were described as the maximal traction stress (the force of failure per surface unit) applied to the glued samples.

#### 2.4.3. Compression Test

As well as assessing the adhesion using the tensile adhesion tests, the compressive strength of the cured glue was also assessed. The cylinder glue samples were prepared by filling a polyethylene mold (9 mm in diameter and height) with the prepared glue, and then left to cure for 10 min before being gently demolded and soaked in a 37 °C PBS bath for 24 h. The compression test was performed (minimum N = 8 for each experiment) on an Instron 5966^®^ device with a load cell of 10 kN and at a displacement speed of 0.5 mm/min until failure. The results were described as the maximal compression stress (the force of failure per surface unit) applied to the glue samples.

#### 2.4.4. Test to Determine Setting Time

Following the ASTM C266-18 standard, the measurement of the setting time of the cement was performed based on the Gillmore needle device (2.12 mm diameter needle with an applied force of 113.4 g and 1.06 mm diameter needle with an applied force of 453.6 g, respectively, for detecting the initial setting and the final setting). The test needle was conducted on the surface of the prepared glue regularly with the abovementioned force. The duration between the beginning of the glue preparation and initial or final hardening of the glue was respectively noted as the initial or final setting time.

#### 2.4.5. Biomineralization Test

The in vivo formation of bone-like apatite on the surface of an implanted material is a prerequisite to osteointegration. This mineralization process can be simulated in vitro by immersion in simulated body fluid (SBF), which was prepared according to the protocol of Kokubo et al. [43]. Glue disks (8 mm in diameter and 2 mm in thickness) were prepared using manual compression casting for 30 min in air for a complete cure in a Teflon mold. After demolding, the glue disks were then immersed in a 37 °C SBF bath, at a volume/volume (SBF/glue disk) ratio of 10:1, for 4 days or 7 days. The SBF bath was fully replaced every 48 h.

After 4- or 7-day immersion, grazing incidence wide-angle X-ray scattering (GIWAXS, Xeuss2.0^®^, Xenocs, Grenoble, France) was performed to identify the crystalline phase on the surface of the glue samples. Then, SEM (SU 5000^®^, Hitachi, Tokyo, Japan) was used to observe the surface morphology and the repartition of mineral substance on the sample.

#### 2.4.6. Cytotoxicity Test

The cytotoxicity of the bone adhesive with pre-osteoblastic MC3T3-E1 cells (ATCC CRL-2593) was assessed with the extraction method according to ISO 10993-5. The cells were seeded at a density of 4 × 10^3^ cells per well in a 96-well plate (Falcon^®^, Merck, Darmstadt, Germany), and were cultivated for 24 h until reaching approximately 60% confluence. Disk samples of the glue (8 mm in diameter and 2 mm in thickness, 240 mg) were prepared in the same way as for the SBF test above, and then each disk was rinsed with 2 mL of serum-free medium for 5 min. Extraction was performed: the disks were immersed in complete culture medium (alphaMEM, Gibco^®^, Thermo Fisher Scientific, Waltham, MA, USA) containing 10% fetal bovine serum and gentamicin at 50 µg/mL, respecting the ratio of 0.2 g/mL, for 24 h at 37 °C in a 5% CO_2_ atmosphere. After 24 h, the extracts were collected. The media in the 96-well plates were completely replaced with 100 µL/well undiluted extracts (100%), and the cells were exposed to the extracts for 24 h. The untreated (negative) control was applied by using the complete culture medium without adhesive disks.

The cellular metabolic activity was measured by alamarBlue^®^ assay, using a 10-fold diluted alamarBlue^®^ solution (Invitrogen^®^, Thermo Fisher Scientific, Waltham, MA, USA) into complete culture media. The cells were incubated for 2 h, and 150 µL/well of medium was measured for intensity of fluorescence at ex. 560 nm/em. 590 nm on a spectrophotometer plate reader (CLARIOstar^®^, BMG Labtech, Ortenberg, Germany). The blank solution (10% alamarBlue^®^ solution) was subtracted from each sample, and the fluorescence intensity data were normalized to the negative control to obtain a percentage representing the “survival rate”.

### 2.5. In Vivo Evaluation of the Efficacy of the Bone Glue for Autograft Fixation

A novel rat model was designed to assess the in vivo biocompatibility of the glue and its efficacy to fix a graft to bone. The ex vivo glued fibula/tibia model described in Section 2.4.2 was the precursor and the basis for the methodology of this in vivo model, in which the tibia supports nearly all of the skeletal load; hence, there is only low mechanical stress from muscular contraction.

#### 2.5.1. Animal Model

The experimental animal procedure, based on the methodology of the ex vivo glued fibula/tibia model, was submitted to and approved by the French Ministry of Education, Teaching and Research and the local Ethical Committee for Animal Experimentation as an animal-use project (No. 26081). The animal housing and surgical procedure were carried out in the Animal Facility of the University of Lille “Département Hospitalo-Universitaire de Recherche Expérimentale (DHURE)”, according to European regulations regarding the protection of animals used for scientific purposes (Directive 2010/63/EU).

Surgery was performed bilaterally on 10 male Sprague Dawley rats (250–300 g, aged 2 months), which were euthanized at two time points: 5 weeks and 12 weeks. Hence, 17 surgical sites were randomly distributed into two groups: the control group without fixation (N = 3 for each time point), and fixation with TTCP/OPS-nPDA glue (N = 7 for each time point).

All surgical operations were performed in aseptic conditions. General inhalation anesthesia was induced in a chamber delivering 4–5% isoflurane/O_2_ gas mixture, and the anesthetic state was maintained with 1.5–2.5% isoflurane/O_2_ gas mixture with an adapted ventilation facemask. Each animal received an intramuscular injection of buprenorphine hydrochloride (0.05 mg/kg) preoperatively to relieve pain.

#### 2.5.2. Surgical Procedure

The rat legs were shaved and scrubbed twice with chlorhexidine solution. Surgery was performed bilaterally with the rats lying in the supine position. A cutaneous incision of the leg was performed next to the anterior face of the tibia. The tibial periosteum was then incised, before tibial and fibular subperiosteal dissection. A 6 mm long segment of the distal fibula was harvested, preserving the proximal fibula, notably the fibular head (avoiding lesion to the fibular nerve). Slight surface grinding of the tibial grafting site (the tibial diaphysis between the angulation and the tibiofibular junction) was performed with a round bur. A thin layer of the prepared glue was firstly applied to the ground surface of the shaft of the tibia, and the fibular ipsilateral autograft was then glued to the tibia under manual compression for 4 min. In the sham group (without glue), the fibular graft was simply deposed on the tibia without any means of fixation. A screw (ø 0.7 × 3.7 mm) was placed 2 mm superiorly to the proximal end of fibula graft as a position indicator for later recognizing any displacement of glued fibula with time. Closure was performed in two planes: muscular and inversed transcutaneous with Vicryl^®^ 4/0. Cutaneous glue Dermabond^®^ (Ethicon^®^, Raritan, New Jersey, United States) was applied to securely close the wound.

Buprenorphine (0.05 mg/kg) was administered intramuscularly twice daily for 3 days for analgesia. The rats were allowed to bear the weight and move freely after waking up.

#### 2.5.3. Euthanasia Procedure and Clinical Evaluation

At weeks 5 and 12, the rats were anesthetized in 5% isoflurane/O_2_ gas mixture and euthanized with an intracardiac injection of 0.3 mL/kg of T61^®^ (MSD, Kenilworth, NJ, USA). The tibia was dissected, explanted, and cross-sectioned at 5 mm proximal and distal to the glued area using a diamond cutting disk.

A macroscopical clinical evaluation was performed focusing on the signs of inflammation or infection surrounding the glued graft, the fixation of the fibular graft vis-à-vis manual traction with a tweezer and the potential displacement of the glued graft referring the position of the graft to the position indicator (screw). Clinical success was defined as an autograft that was well fixated (i.e., withstanding the manual traction) and without any displacement, and was noted for calculating the success rate of the group.

The explanted samples were then placed in 10% neutral buffered formalin for 24 h until ready for analysis.

#### 2.5.4. Microcomputed Tomography (microCT) Evaluation

The explanted tibia samples were examined by microCT (Skyscan^®^ 1172, Bruker microCT, Kontich, Belgium) and set with the following parameters: voltage 100 kV, current 100 μA, image rotation 0.1500, pixel size 19.99 μm. Multiplanar slices were reconstructed with NRecon software (Bruker microCT, Kontich, Belgium). Beam hardening, notably on metallic hardware, was reduced with the reconstruction software. The tibia–adhesive–fibula interfaces were qualitatively evaluated by observing the space between the bone and the adhesive.

#### 2.5.5. Histological Evaluation

Since the decalcification process would indistinctly affect the bone tissue and the mineralized adhesive, which would lead to potential analysis difficulties, the harvested samples (immersed in 70% ethanol) were sent to LLS Rowiak LaserLabSolutions (Hannover, Germany) for non-decalcified preparation. The samples were dehydrated in a graded series of alcohol and embedded in PMMA. The PMMA block was then cut into 10 μm thick slices with a laser microtome (TissueSurgeon LLS Rowiak, Hannover, Germany). The slices were colored with hematoxylin and eosin stain.

### 2.6. Statistics

Statistically significant differences in the quantitative data were analyzed using the BiostaTGV website developed by the Institut Pierre Louis d’Epidemiologie et de Santé Publique affiliated with Sorbonne University, Paris, using a Mann–Whitney non-parametric test. The risk α was set at 5%.

## 3. Results

### 3.1. Characterization of Synthesized nPDA

**Figure 1:**
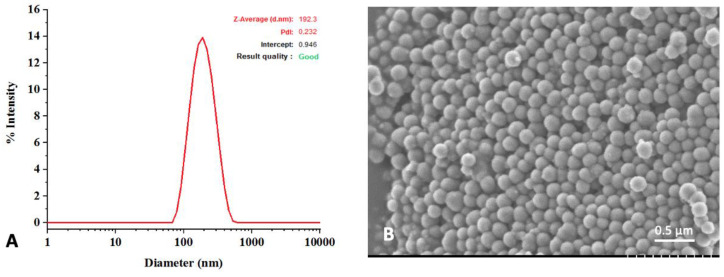
Characterization of nPDA with diffusion light scattering showing an average diameter of 190 nm and a polydispersity of 0.23 (**A**), and with scanning electron microscopy showing spherical particles uniformly distributed (**B**).

The dynamic light scattering analysis of the nPDA showed an average diameter of 190 nm and a polydispersity of 0.23, demonstrating that nPDA has a clearly narrow size distribution (Figure 1A). Scanning electron microscopy image confirmed that the nPDA has a spherical morphology and is uniformly distributed (Figure 1B).

### 3.2. Impact of Glue Formulation on Adhesion

#### 3.2.1. Instrumental Tensile Adhesion Test for Formulation Screening and Optimization

The molar percentage (%_mol_) of OPS in the TTCP/OPS mixture, the liquid-to-powder ratio (mL/g), and the mass percentage (%_wt_) of the added nPDA were screened in order to select an optimal formulation for further characterization.

**Figure 2:**
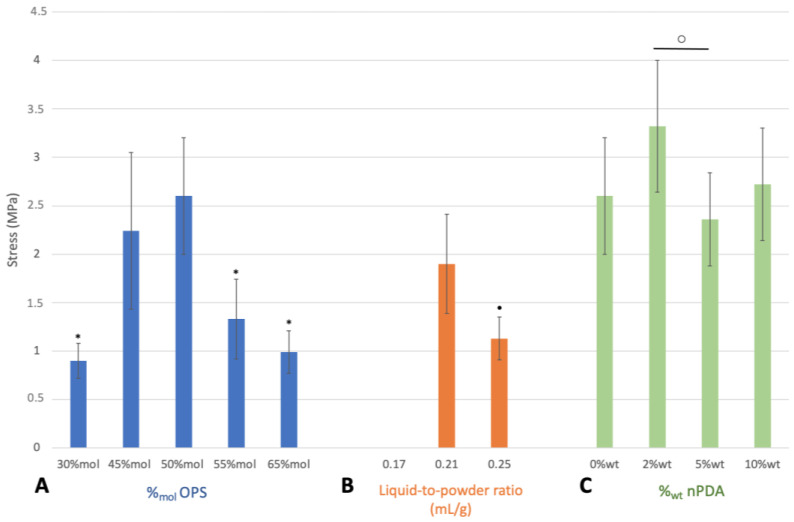
Instrumental tensile adhesion test using titanium cylinder samples evaluating adhesion after 1 h immersion in a 37 °C PBS bath in order to optimize the formulation of the bone glue by varying: (**A**) the molar percentage (%_mol_) of OPS in the TTCP/OPS mixture, (**B**) the liquid-to-powder ratio (mL/g), and (**C**) the nPDA content (%_wt_). A statistically significant difference was shown: in (**A**) by * (*p* < 0.05) compared to the group “50%_mol_OPS”, in (**B**) by “•” (*p* = 0.016) compared to the group “liquid-to-powder ratio 0.21 mL/g”, and in (**C**) by “○” (*p* = 0.013) between the marked groups.

Firstly, the optimal molar percentage of OPS in the TTCP/OPS mixture associated with the strongest adhesive force (2.6 ± 0.6 MPa) was found at 50%_mol_ OPS (Figure 2A). Indeed, with the increase in OPS%_mol_ in the TTCP/OPS mixture above 50%_mol_, the adhesive strength of the glue decreased progressively to 1.33 ± 0.41 MPa (*p* = 0.0019) at 55%_mol_ and 0.9 ± 0.18 MPa (*p* = 0.0009) at 65%_mol_. Similarly, when the OPS%_mol_ decreased below 50%_mol_, the adhesive strength of the glue also decreased progressively to 2.24 ± 0.81 MPa (*p* = 0.293) at 45%_mol_ and 0.99 ± 0.22 MPa (*p* = 0.015) at 30%_mol_. Moreover, at 30%_mol_ OPS, the setting time of the glue also turned out to be too short to work with. These results are in accordance with previous work dealing with the preparation of CPC/OPS bone glue (OsStic^TM^) [16]. Although the role of OPS in OPS/CPC glue has not yet been clearly proven, the hypothesis, as suggested by Kesseli et al. [44], is that OPS may participate in a classic acid-based reaction evolving crystalline calcium phosphoserine monohydrate, which temporarily precedes the competing formation of hydroxyapatite via the hydrolysis of the TTCP. Thus, there must be an optimal concentration of OPS for this reaction, more or less than which will lead to the redundant OPS or uncomplete reaction and reduce the adhesion power of the glue.

Afterwards, as can be seen in Figure 2B, the optimal liquid-to-powder ratio was found at 0.21 mL/g to reach the highest adhesion force of 1.9 ± 0.51 MPa. In fact, beyond this, when the liquid-to-powder ratio increased (0.25 mL/g) or decreased (0.17 mL/g), the adhesive force of the glue significantly decreased to 1.13 ± 0.22 MPa (*p* = 0.016) or was lost (no data recorded), respectively. This is consistent with the results obtained in previous studies on TTCP-based bone adhesives (Tetranite^®^) [15,18]; however, is not consistent with the results found for αTCP-based bone adhesives (0.15 mL/g) [16]. This difference might be explained by the different reactivity of CPC; αTCP is considerably more reactive in a humid environment than TTCP due to its high specific energy [45], therefore requiring a lower volume of liquid to cure.

Finally, as shown in Figure 2C, the optimal weight percentage of the nPDA was found at 2%_wt_ with the strongest adhesion strength (3.32 ± 0.68 MPa), compared to that at 0%_wt_ (2.6 ± 0.6 MPa, *p* = 0.08), 5%_wt_ (2.36 ± 0.48 MPa, *p* = 0.013), and 10% _wt_ (2.72 ± 0.58 MPa, *p* = 0.15).

Based on the above results, the mixture composed of 50%_mol_TTCP/50%_mol_OPS-2%_wt_nPDA, associated with a liquid-to-powder ratio of 0.21 mL/g, formulated an optimal glue, resulting in the best adhesive performance. This formulation was thus applied in further experiments comparing the glue without nPDA (50%_mol_TTCP/50%_mol_OPS).

#### 3.2.2. Impact of Immersion Time on Adhesion of Optimal Glue Formulation

**Figure 3:**
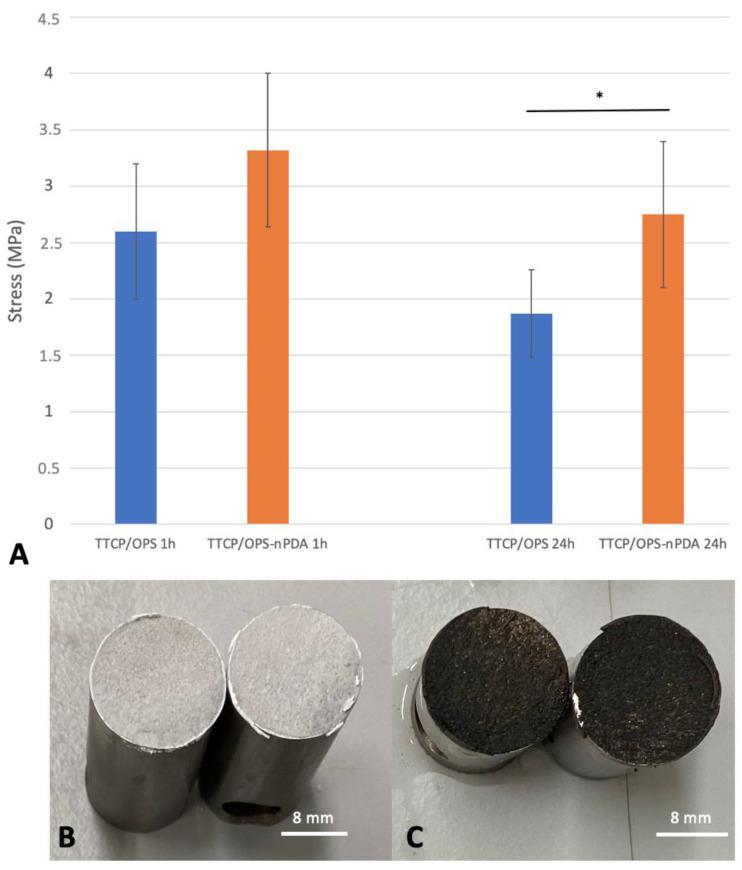
Instrumental tensile adhesion tests using titanium cylinder samples comparing TTCP/OPS glue versus TTCP/OPS-nPDA glue: (**A**) maximum traction stress after 1 h and 24 h immersions in a 37 °C PBS bath. * stands for a statistically significant difference (*p* = 0.0029) between two marked groups; surfaces of titanium samples glued with TTCP/OPS (**B**) or with TTCP/OPS-nPDA (**C**) after rupture, mostly showing a cohesive failure for both.

A bone adhesive in clinical use must be able to form a bond to bone under physiological conditions, i.e., humid environment and homeothermy of 37 °C. Therefore, the wet environment adhesion of the glue was investigated by immersing the glued titanium samples in a 37 °C PBS bath for various durations, as demonstrated in Figure 3A. In the TTCP/OPS group, the adhesive strength decreased significantly (*p* = 0.012) with the increased duration of immersion in PBS: 2.6 ± 0.6 MPa at 1 h versus 1.87 ± 0.39 MPa at 24 h, while in the TTCP/OPS-nPDA group, it decreased insignificantly (*p* = 0.06): 3.32 ± 0.68 MPa at 1 h versus 2.75 ± 0.65 MPa at 24 h. The addition of nPDA seems to enhance the stability of the adhesive performance in a humid environment during a prolonged 24 h immersion. Moreover, under the same conditions, the TTCP/OPS-nPDA glue tended to show a superior adhesive force on titanium cylinders compared with the TTCP/OPS glue: after a 1 h immersion in PBS, 3.32 ± 0.68 MPa versus 2.6 ± 0.6 MPa, respectively (*p* = 0.083); after a 24 h immersion, 2.75 ± 0.65 MPa versus 1.87 ± 0.39 MPa, respectively (*p* = 0.0029). These implied the better long-term adhesive property of the bone glue thanks to the integration of nPDA.

In addition, the fractography and failure analysis (Figure 3B,C) mostly showed a cohesive failure (i.e., fracture occurred in the bulk of the adhesive), which means that the adhesive interactions between the adhesive and the titanium surface are superior to the cohesive forces (i.e., the force of attraction within the adhesive). Such a failure profile is a sign of a strong adhesive property and is in accordance with previous reports of organo-mineral adhesive [15,16].

### 3.3. Characterization of Glue Performance with Optimal Formulation

#### 3.3.1. Adhesion to Bovine Bone: Ex Vivo Instrumental Tensile Adhesion Test

**Figure 4:**
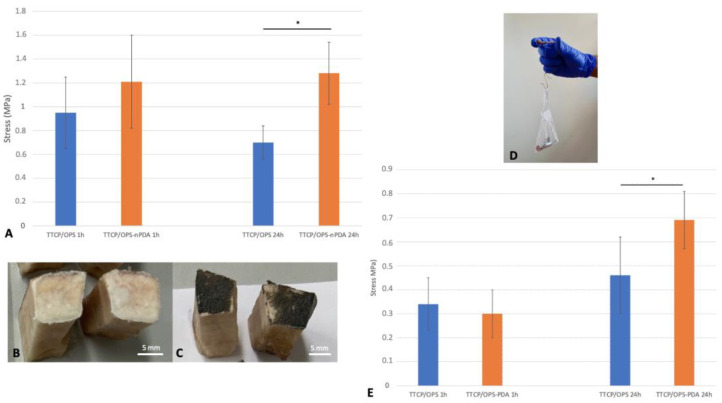
Ex vivo tensile adhesion test comparing TTCP/OPS glue versus TTCP/OPS-nPDA glue after 1 h and 24 h immersions in a 37 °C PBS bath: (**A**) instrumental tensile adhesion test using cuboid bovine cortical bone samples. * stands for a statistically significant difference (*p* = 0.00093) between two marked groups; surfaces of bone samples glued with TTCP/OPS; (**B**) with TTCP/OPS-nPDA; (**C**) after rupture, mostly showing a cohesive failure for both; (**D**) experimental setup of manual tensile adhesion test on the glued rat tibia/fibula: a fibula segment sample (autograft) was glued to the tibia by a thin layer of the prepared glue, between which a Vicryl^®^ 4/0 suture thread was deposed perpendicular to the anatomical axis of the tibia and glued together. An evaluation of the adhesive strength was performed by traction with progressively increasing standard weights attached to the Vicryl^®^ thread (minimum N = 7 for each experiment) until rupture of adhesion. The sum of the applied weights was thus noted as the traction force of failure; (**E**) maximum traction stress of manual tensile adhesion test of glued fibular/tibial bone. * stands for a statistically significant difference (*p* = 0.029) between two marked groups.

The comparison of the two glue formulations (w/o nPDA) ex vivo through instrumental tensile adhesion tests with cuboid bovine bone samples after immersion in PBS is presented in Figure 4A. Similar to the test above with the titanium samples, the adhesive strength of TTCP/OPS-nPDA to bovine bone seemed superior to that of TTCP/OPS: at 1 h, 1.21 ± 0.39 MPa versus 0.95 ± 0.3 (*p* = 0.247); at 24 h, 1.28 ± 0.26 MPa versus 0.7 ± 0.14 MPa (*p* = 0.00093). Regarding the impact of the duration of immersion in PBS, from 1 h to 24 h, the adhesive force tended to decrease (*p* = 0.115) for the TTCP/OPS group, but increased (*p* = 0.674), contrastingly, for the TTCP/OPS-nPDA group, suggesting the better adhesive performance of the latter. It is also noticed that, under the same conditions, the mean values of rupture strength for the titanium samples were generally higher than those for the bone samples. This could be attributed to the higher affinity for titanium, which was already shown in a previous study [15].

The macroscopic examination of failure analysis after rupture mostly showed a cohesive rupture (Figure 4B,C), which is similar to that observed above with the titanium samples.

To sum up, thus far, TTCP/OPS-nPDA glue has shown better wet environment adhesion to both titanium substrate and bovine cortical bone than TTCP/OPS glue.

#### 3.3.2. Adhesion of Glued Rat Tibia/Fibula: Ex Vivo Manual Tensile Adhesion Test

The ex vivo model of rat fibula graft glued to tibia (Figure 4D) was designed to mimic the clinical scenario of an autogenous bone grafting. The results of the adhesive force from the manual tensile adhesion tests achieved for the glued rat tibia/fibula (Figure 4E) showed, after a 1 h immersion in a PBS bath, no difference between the two formulations (*p* = 0.522): 0.3 ± 0.1 MPa for TTCP/OPS-nPDA versus 0.34 ± 0.11 MPa for TTCP/OPS. Nevertheless, after 24 h of immersion, the adhesive force increased for both TTCP/OPS (*p* = 0.335) and TTCP/OPS-nPDA (*p* = 0.0021), and the performance of TTCP/OPS-nPDA (0.69 ± 0.12 MPa) also became significantly (*p* = 0.029) superior to that of TTCP/OPS (0.46 ± 0.16 MPa). The examination of the fracture surface mostly showed a mixed cohesive and adhesive failure, which could be due to the lower congruence of surfaces to glue compared to previous assays.

To summarize, these results are consistent with previous results obtained from ex vivo test models carried out with bovine bone. Nevertheless, this rat tibia/fibula model evaluated by manual traction, with a setup of autograft fixation, is much closer to a clinical setting. Overall, after 24 h in wet environments (a 37 °C PBS bath), both the in vitro and ex vivo results clearly demonstrated the advantage of the TTCP/OPS-nPDA formulation over that without nPDA.

#### 3.3.3. Cohesive Strength of the Glue: Instrumental Compression Test

**Figure 5:**
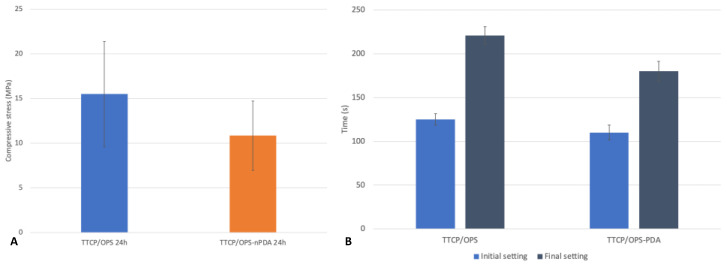
Characterization of the bone adhesive TTCP/OPS versus TTCP/OPS-nPDA: (**A**) compression test using cylinder glue samples after 24 h immersion in a 37 °C PBS bath (*p* = 0.083); (**B**) measuring the setting time by Gillmore test on disk glue samples.

As illustrated in Figure 5A, there is no statistically significant difference (*p* = 0.083) in the compressive strength between the TTCP/OPS (15.5 ± 5.91 MPa) and TTCP/OPS-nPDA (10.85 ± 3.88 MPa). The addition of nPDA seemed not to affect the cohesive strength of the glue. Indeed, Liu et al. [34] found an enhanced compressive strength of a brushite-based calcium phosphate cement cured by adding a suspension of PDA. However, the composition of our adhesive (TTC/OPS) is rather different from that of the cement of Liu et al., and nPDA seems not to impact the reaction pathways in TTCP-OPS organo-mineral systems, and thus does not change the compressive strength of the glue.

Nevertheless, the brittleness is known as the inherent characteristic of ceramic materials responsible for the spread of microfissures and subsequent fractures of the material under mechanical stress. The reinforcement of such bone adhesives are critical for their successful application in vivo. Various approaches of organo-mineral bone adhesive reinforcement were thus studied, such as the addition of fibers to stop the spread of microfissures. For example, the addition of resorbable polymers such as poly(lactic-co-glycolic acid) (PLGA) fibers or chitosan lactate or PLGA sutures in the Tetranite^®^ glue was assessed [18], and showed increased compressive strength with a reduced adhesive property. Therefore, fiber-reinforced TTCP/OPS-nPDA glue could be a solution to enhance the compressive strength of the glue, but this requires further research.

#### 3.3.4. Setting Time

Setting time is important to assess as it helps to define the working time, that is, the interval between the mixing time (the beginning of the chemical reaction in the adhesive) and the initial setting time. The results of the Gillmore test between the TTCP/OPS glue and the TTCP/OPS-nPDA glue, as detailed in Figure 5B, showed no statistical difference, either in the initial setting of 125 ± 6.5 s versus 110 ± 8.5 s (*p* = 0.08) or in the final setting of 221 ± 10 s versus 180 ± 1.5 s (*p* = 0.08). In a clinical setting of fracture reparation, sufficient working time is needed not only to apply the glue onto the bone fragments, but also to place them correctly to restore their proper alignment. If this setting time is too long, the delayed hardening of the glue may perturb the maintenance of reduction. Moreover, it should not be longer than that of the current method of fixation, as a longer surgical operation increases the risk of postoperative complications [46,47]. The ISO 5833 considers it acceptable if the initial setting lasts a few minutes and the final setting ends in less than 10 min [8,48]. According to this, the setting time of the developed glue is suitable for application and bone fragment placement and is quick enough compared to the current fixation methods.

#### 3.3.5. Biomineralization Test

**Figure 6:**
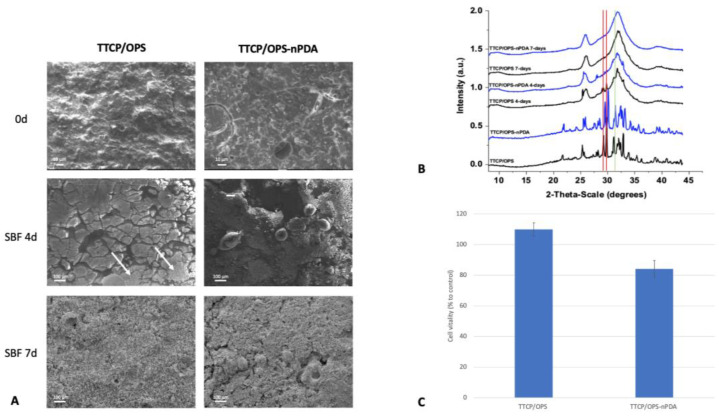
In vitro biological reactivity tests. Biomineralization test by soaking glue disks in simulated body fluid (SBF), with SEM images (**A**) showing: full mineralization layer on the TTCP/OPS-PDA surface after 4 days, with only a few small patches on TTCP/OPS disks (white arrow); after 7 days, a more abundant layer of mineralization on TTCP/OPS-nPDA disks and only a fine layer of apatite on TTCP/OPS disks; (**B**) grazing incidence wide-angle X-ray scattering (GIWAXS) analysis of glue disks after 0-, 4-, and 7-day immersion in SBF showing the hydroxyapatite phase in the mineralized layer (red bar for indicating the characteristic peaks of TTCP and green bar for indicating the characteristic peak of hydroxyapatite); (**C**) cytotoxicity test (extraction method) carried out on TTCP/OPS and TTCP/OPS-nPDA glue disks with MC3T3-E1 pre-osteoblast cells by alamarBlue^TM^ assay (*p* = 0.0009). Data were normalized against the 100% negative control (cells cultured in complete culture medium without adhesive extract) to obtain a percentage of relative cell vitality representing the “survival rate”.

This test aims to reproduce, in vitro, the biomimetic mineralization occurring at the surface of material in vivo when a material is implanted close to bone tissue [49]. Therefore, it predicts the behavior of a material with bone tissue. As depicted in Figure 6A, the surface morphology of the glue disks, examined by SEM, showed that after a 4-day soak in SBF, the surface of the TTCP/OPS-nPDA disk was largely covered by a bone-like apatite layer, whereas there were only a few patches on the TTCP/OPS sample. After a 7-day immersion, the TTCP/OPS was eventually covered by a fine layer of apatite, whereas the TTCP/OPS-nPDA was already covered by an abundant layer of mineralization.

The crystalline phases of this mineralization on the surfaces of all disks were identified to be hydroxyapatite (HA) using the structural measurement technique GIWAXS (Figure 6B). Indeed, the GIWAXS patterns show that the main phases were TTCP, with two characteristic peaks at θ = 28–29 degrees, on the surface before soaking (day 0). While the TTCP diffraction peaks on both TTCP/OPS and TTCP/OPS-nPDA disappeared after soaking in SBF on day 4 and day 7, a characteristic HA peak at θ = 32 degrees appeared and gradually increased over time [50,51].

Therefore, once soaked in SBF, a hierarchical bone-like apatite layer can be formed and developed in a time-dependent manner on the surface of both glues. SEM images clearly showed that the incorporation of nPDA can accelerate the mineralization process of TTCP/OPS-nPDA and thus increase its bioactivity toward bone tissue at an early stage, owing to the well-known “PDA-assisted HA formation” (allowing the concentration of calcium ions at the interface and promoting HA mineral nucleation) [52].

#### 3.3.6. Cytotoxicity Test

After exposure to TTCP/OPS or TTCP/OPS-nPDA glues, the in vitro cytotoxicity assay carried out with MC3T3-E1 pre-osteoblast cells showed (Figure 6C) a cell survival rate of 110% ± 3% and 85% ± 5%, respectively. These results thus confirmed the absence of cytotoxicity in both adhesive formulations according to the threshold of 70% set by ISO10993-5. This observation is in agreement with a recent report of the excellent cytocompatibility [22] and biocompatibility of organo-mineral adhesives in vivo [15,18,23].

Indeed, the good cytocompatibility of nPDA (at concentrations below 0.05 mg/mL) with multiple cell lines [42,53,54,55] and its biocompatibility in vivo [56,57,58] have been well reported in the literature. It is therefore no surprise that TTCP/OPS-nPDA glue possesses no cytotoxicity, with a cell survival rate of 85%. Nevertheless, we do notice the slightly lower cell survival rate compared to TTCP/OPS glue, which could be attributed to the release of the remaining non-oxidized dopamine or self-assembled trimer of dopamine_2_/DHI from the nPDA material [59,60].

### 3.4. In Vivo Evaluation

So far, TTCP/OPS-nPDA adhesive has demonstrated superior adhesive properties in vitro and ex vivo compared to TTCP/OPS, and no cytotoxicity in vitro; thus, an in vivo assessment of its biocompatibility and adhesive efficacy is highly required. The rat glued fibula/tibia (autograft fixation) model was applied under two experimental conditions: the absence of fixation (control group) and the fixation of the fibular graft with TTCP/OPS-nPDA glue.

#### 3.4.1. Clinical Observation

The rats who survived were healthy up to termination. The clinical success of the fibular graft fixation (stably stuck onto the tibia without secondary displacement) was assessed after they were euthanized, and these findings are summarized in Table 1.

In the control group, the grafts were lost due to the absence of fixation, and therefore could not be retrieved in the rats who were euthanized after 5 or 12 weeks. Indeed, movement between bone fragments prevents bone healing and leads to graft failure; thus, fixation is necessary [61,62]. Even if the tibia bears nearly all of the skeletal load, slight mechanical stress from the contraction movements of the surrounding muscular tissues is still applied onto the graft. This mechanical stress, although low, prevented bone healing and subsequently failed. Therefore, the designed model is validated for evaluating the efficacy of any means of autograft fixation.

**Figure 7:**
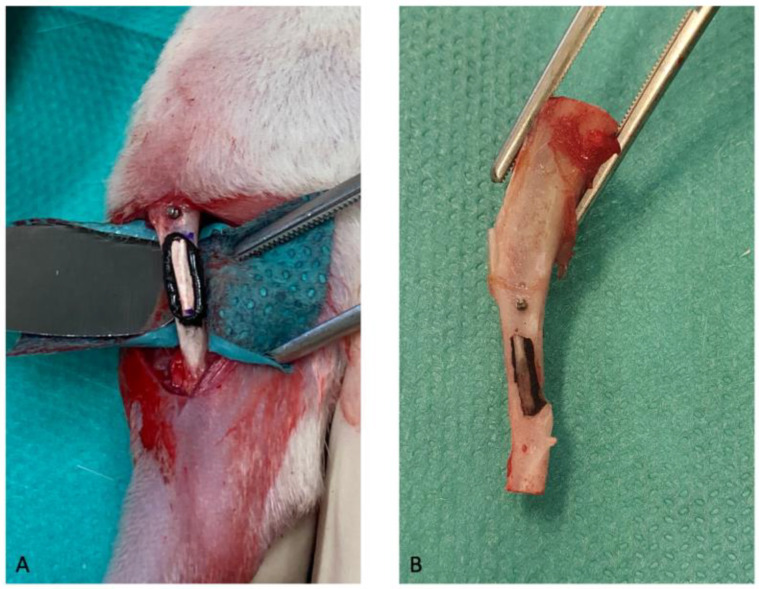
Photographs of in vivo rat model with glued fibula/tibia (autograft fixation) used for evaluating the fixation of the fibular graft with TTCP/OPS-nPDA glue: (**A**) intraoperative photograph showing a fibular autograft glued to a rat tibia with a position indicator (screw); (**B**) photograph of a fibular autograft glued to a rat tibia after 12 weeks, which remained stable and was without secondary displacement.

In the TTCP/OPS-nPDA group, six out of seven grafts (86%) remained stable without secondary displacement at 5 weeks, and five out of seven (71%) had clinical success at 12 weeks (shown in Figure 7). Therefore, the TTCP/OPS-nPDA glue was an effective means of fixation for the autograft.

#### 3.4.2. MicroCT Evaluation

**Figure 8:**
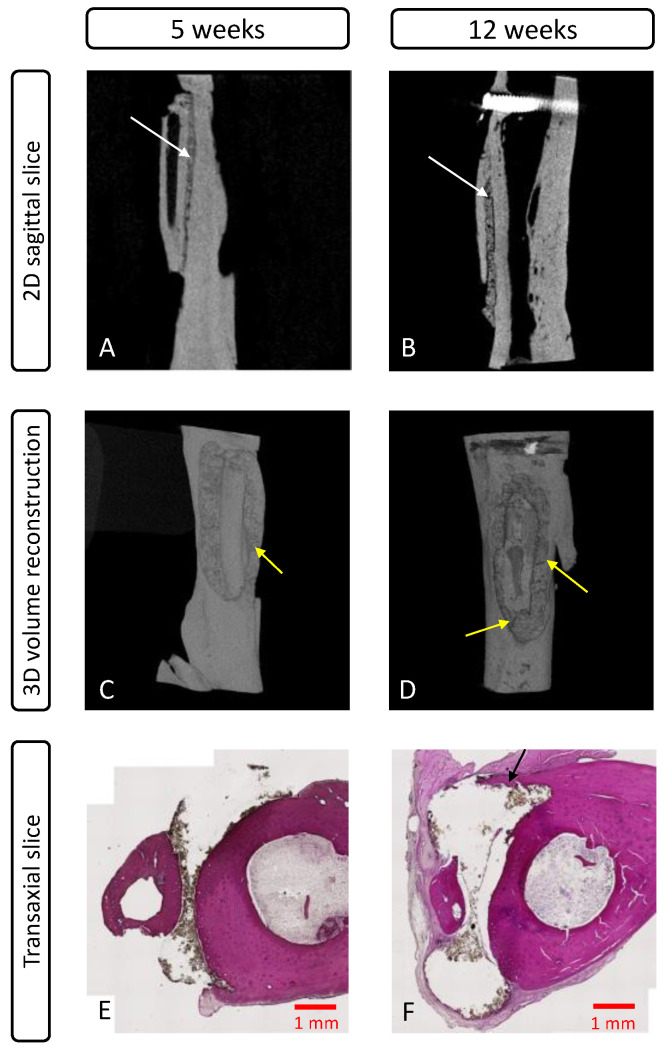
Evaluation in vivo of glued rat fibula/tibia with TTCP/OPS-nPDA glue at 5 or 12 weeks: microCT images of 2D sagittal slices (**A**,**B**) and 3D volume reconstruction (**C**,**D**) of a stably fixed fibular graft on a rat tibia showing a continuity of the interface between the graft, the adhesive, and the tibia (white arrows), with some rare fissures in the adhesive indicated by yellow arrows (**C**,**D**); non-decalcified histological sections (**E**,**F**) of glued fibular autograft to tibia with clinical success stained with hematoxylin and eosin (×10) showing the absence of inflammation and the coverage of newly formed bone over the TTCP/OPS-nPDA glue at 12 weeks ((**F**), black arrow).

No microCT acquisition was performed for the control group, as there was no sample with a stable graft. Analysis performed on the TTCP/OPS-nPDA samples (Figure 8) corroborated the above findings in clinical assessments: all cases with clinical success showed a continuity (without space) of interface between the graft, the adhesive, and the tibia at both 5 and 12 weeks, which could be associated with the clinical success of the graft.

#### 3.4.3. Histological Evaluation

A qualitative histological assessment was performed on non-decalcified samples in order to avoid adhesive dissolution and subsequent autograft loosening and help to better visualize the graft–adhesive–tibia interface. No histological analysis was performed on the control group, as there were no samples with a graft on the tibia. For the sample with clinical success in the glue group, the analysis at 5 (Figure 8E) and 12 weeks (Figure 8F) showed no signs of inflammation surrounding the adhesive (black-brown color typically for nPDA) between the glued bone surfaces, and the presence of adhesive at 12 weeks implies its slow degradation. In particular, a newly formed bone tissue coverage over the glue was observed at 12 weeks (Figure 8F), demonstrating the bone regeneration-promoting property of the adhesive. This could be attributed to the well-known potential of PDA for generating a microenvironment adapted to osteoblastic adhesion/proliferation, thus promoting the osteogenesis [34].

It can also be noted that due to the technical limitations related to the laser-based section technology, the internal structure of the adhesive and the eventual cellular colonization within the adhesive unfortunately could not be visualized (Figure 8E,F). In fact, nPDA, which is structurally close to melanin, possesses the same photoprotective abilities, i.e., it absorbs a wide array of electromagnetic rays [42,53], including the wavelength of the laser used for a section of the sample. Hence, in most cases, the obtained sections were not cut completely. Indeed, the cutting of hard tissue is always more troublesome than for soft tissue; some alternative techniques, such as a microtome with specific blades or ground sectioning, could be very traumatic to the sample—even more so than the laser section method.

In the clinically failed cases, the encapsulation of the adhesive with fibrous tissue was systematically found. Such failures may have multiple causes, but the presence of fissures within the adhesive may explain why the process of integration was observed in some parts of the adhesive, while the inflammatory reactions were observed in other parts (with fissures). These fissures are related to the intrinsic brittleness of CPC, and the reinforcement of the mechanical properties of the adhesive, as mentioned previously, may help to prevent such a failure.

## 4. Discussion

Adhesive has gained increasing popularity due to its handling versatility and the important advantages it has over other joining methods. An ideal bone adhesive has to face major obstacles such as a wet environment, the multiscale surface roughness of bone tissue, and biosafety, among many others. In this study, biomimetic approaches have been applied to design a bio-based bone adhesive combining the well-known CPC with OPS (inspired by sandcastle worms) and PDA chemistry (inspired from both mussels and sandcastle worms).

Notably, the catechol-rich nPDA [34,35,36] was added to strengthen the adhesive properties of a TTCP/OPS-based adhesive and to promote HA nucleation and osteogenesis. Both the in vitro and ex vivo tensile adhesion tests achieved in this study have confirmed that nPDA played such a role in enhancing the bond to bone and titanium of the TTCP/OPS-nPDA glue compared to TTCP/OPS glue in a simulated physiologic environment. Several mechanical testing methods for evaluating the adhesive strength of bone adhesives in vitro/ex vivo have been described [15,16,18,19,37,38,39,40], showing a wide range of bone bond strength values depending on the type of bone as well as the bone condition and surface preparation. It is worth noting that our experimental conditions were set very “unfavorably” for adhesive bonding, such as a flat surface without any imbrication, the absence of pretreatment, and in a wet environment, all of which were set to simulate the in vivo setting in order to obtain the most reliable data on the adhesive properties. We would like to compare these resultant values (1.2–1.4 MPa with bone) with those of the literature (often claiming up to 1.5–2 MPa with bone [15,16]); however, with our very “unfavorable” experimental conditions, such a comparison is difficult and irrational. Overall, our results obtained from the novel-designed rat model confirmed that such an adhesive could be used either on its own to stick bone fragments together, or potentially to enhance the primary stability of titanium implants such as dental implants, or in conjunction with titanium fixation hardware in cases where load-bearing reconstruction is necessary.

As well as the adhesive property, a bone adhesive can ideally promote bone formation in order to obtain ad integrum bone healing without any artificial implant interposition. The TTCP in the adhesive of this study, as with other CPCs, exhibits well-known osteoconductive properties [45,63]. OPS, a phosphorylated amino acid, is abundant in bone extracellular matrix proteins such as osteopontin, bone sialoprotein, and osteocalcin, and is responsible for promoting osteoblastic differentiation [64], inducing the nucleation of HA and enhancing bone formation [65]. Thus, the adhesive-coordinated network of “calcium phosphoserine monohydrate complex”, formed by mixing OPS and TTCP with water, can initiate bone calcification [44]. However, the role of nPDA addition to CPC/OPS cements is definitely needed to further investigate in depth, e.g., the reaction and products of this new mineral–organic system to identify the factors contributing to the adhesive strength of the system. Moreover, nPDA is known to be osteoinductive by generating a microenvironment adapted to promote osteogenesis [34], which was demonstrated in the biomineralization test (SBF test), in which there was a more rapid and abundant mineralization on the TTCP/OPS-nPDA than on the TTCP/OPS glue. TTCP/OPS-nPDA, therefore, combines all pro-osteogenic properties of each of the above components.

In vivo evaluation is an important step towards human trials, which have been recognized as a major hurdle on the path from bench to bedside. Sterizability, which is a prerequisite, has been assessed (Supplementary materials). Animal models, specifically for assessing the adhesive efficacy of bone adhesives, are barely found in the literature. A recent systemic review [41] focusing on the in vivo evaluation of synthetic bone adhesive materials found that: (i) rabbits were the most frequently used species, followed by rats, (ii) long bones were the most frequently studied regions, and (iii) burr hole defect or cortical perforation models were the most frequently used for assessing bone glue.

Even so, the process of bone remodeling in rats is very similar to the Haversian remodeling found in humans [66], and it is still the most commonly used species [67] if considering in vivo models of bone repair in general. Therefore, we chose the rat model in this study. Regarding the design of model, a burr hole defect is indeed perfectly suitable for assessing a bone substitute material, but not a bone adhesive. An adhesive has to bind two fragments together and resist separation under mechanical stress, not fill the gap in a load-free hole defect. Currently existing organo-mineral adhesives, such as Tetranite^®^ and OsStic^™^, were mostly evaluated in animal models without mechanical stress [18,20,24]; hence, the results cannot be extrapolated to the clinical situation with mechanical stress. Since any movement between bone fragments prevents bone healing, the rigid fixation of autografts is essential for a successful bone graft [61,62]. Under this context, for the first time, an in vivo autograft fixation model was designed and applied in this study, where the graft is affixed on the tibia surface and will be subjected to low mechanical stress from the contraction movements of the muscles surrounding it since the tibia supports nearly all of the skeletal load [68]. We demonstrated that TTCP/OPS-nPDA adhesive successfully fixed the autograft without secondary displacement in the majority of the studied cases, which confirms the excellent adhesion to bone tissue under low stress. In addition, the addition of nPDA to the adhesive seems to promote osteogenesis by covering new bone tissue over the TTCP/OPS-nPDA adhesive. Indeed, we are also aware of the limits of this rat model, which could not demonstrate the performance of the bone glue in terms of clinical fracture. Therefore, more efforts are required to develop new ex vivo/in vivo models for reaching such a requirement.

As well as reinforcing the adhesive and osteoinductive properties of the bone adhesive, nPDA could also be functionalized with various biomolecules, which can be adsorbed in the network of nPDA by π–π stacking, hydrogen bonding, and felectrostatic and hydrophobic interactions [69], thus conferring the bioactive properties to adhesives. Bone infection is a common complication of bone fractures, especially open ones [70], and may also occur following any surgery such as osteotomies [71,72] or autograft placement [73], even when respecting strict aseptic conditions. The poor defense of bone tissue against infections, in addition to the poor penetration of systemic antibiotics into bone tissue, explains the difficulties in treating bone infections [74] and consequent non-union. Therefore, the local delivery of antimicrobial drugs through bone adhesives may help prevent infections along with bone fixation. Various antimicrobial agents could be considered, such as antibiotics, metal ions, quaternary ammonium salts, and nitric oxide, which have all been loaded into nPDA to generate antibacterial functional materials [75]. Apart from anti-infection, other biofunctions such as promoting bone healing and bone growth can be targeted by loading nPDA with molecules such as bone morphogenetic protein 2 (BMP2) [76], adenosine [77], osteogenic peptides [78], and simvastatin. nPDA would therefore offer various possibilities for the addition of new function to the TTCP/OPS-nPDA adhesive.

## 5. Conclusions

To conclude, the novel bio-inspired TTCP/OPS-nPDA adhesive showed superior adhesion to bone ex vivo and titanium in vitro compared to TTCP/OPS. The in vivo evaluation confirmed the biocompatibility of this adhesive and its efficiency for bone fixation under low mechanical stress in an autograft fixation model. The in vivo evaluation in a model under high mechanical stress such as a bone fracture setting will thus be pursued. Finally, nPDA may also serve as a platform for biofunctionalization, e.g., conferring an antibacterial property to the adhesive.

This was the first study to focus on this bone adhesive formulation. Subsequent studies are needed to strengthen the motivation behind the addition of nPDA to CPC/OPS cements, e.g., by investigating the reaction and products of this mineral–organic system to identify the factors contributing to the adhesive strength of the system, performing ex vivo biomechanical testing in other models (bending or torsion test) to better demonstrate the adhesion of the bone glue to comminuted fractures, and investigating the degradation/dissolution of the system in physiological fluids in the long term.

## Figures and Tables

**Table 1:** Results of clinical assessment of the autograft fixation with success.

	No Fixation	TTCP/OPS-nPDA
5 weeks	0% (0/3 sites)	86% (6/7 sites)
12 weeks	0% (0/3 sites)	71% (5/7 sites)

## Data Availability

Data available on request due to restrictions, e.g., privacy or ethics.

## Supplementary Materials

The following supporting information can be downloaded at: https://www.mdpi.com/article/10.3390/pharmaceutics15041233/s1.
