# Supplementary material for: In Vitro and In Vivo Evaluation of a Bio-Inspired Adhesive for Bone Fixation
Source: Pharmaceutics. 2023 Apr 13;15(4):1233. doi: 10.3390/pharmaceutics15041233 (PMC10146643; doi:10.3390/pharmaceutics15041233)
Supplement: Supplementary file 1 [file pharmaceutics-15-01233-s001.zip › pharmaceutics-2273605-supplementary.pdf]

Supplemental information

Impact of sterilization on the property of the optimized glue – Material & Method

Sterilization process

Sterilization is a prerequisite for any implantable material. It must be assessed whether the sterilization process ensures sufficient sterilization without altering the material properties. The powders of TTCP, OPS and nPDA were sterilized separately by two sterilization methods for comparison: γ irradiation (40 kGy) and ethylene oxide (EtO). Following the ISO 11137 standard, γ irradiation was performed at 40 kGy; EtO sterilization was performed with the following parameters: gas concentration 500 mg/L, 43°C, relative humidity 60%, and exposure time 5 hours. Both sterilization procedures were performed by Cousin Biotech company (Wervicq-Sud, France).

Traction test

The impact of the sterilization of glue powder on the adhesive strength of glue was evaluated by traction test, similarly as described in 2.3.2, using titanium cylinders on the Instron 4466 device. Results were then compared with those of the non-sterilized powders and between two sterilization processes.

Sterility test

To confirm sterilized glue powders do not contain viable microorganisms, microbiological test for sterility was performed according to WHO QAS/11.413 standard to validate the sterilization procedure. Briefly, 100 mg of different powders (TTCP, OPS, and nPDA), treated by γ irradiation or EtO, were each put in 10 mL of brain-heart infusion (BHI) broth and incubated at 37°C. At 1, 7, 14 and 21 days, after careful homogenization, 0.1 mL of

the powder/BHI was taken from each tube, and smeared on an agar BHI plate. The agar BHU plate was then incubated 24 hours at 37°C. Twenty-four hours later, the agar BHI plate was observed to assess if any bacterial colony was growing.

Impact of the sterilization on the properties of bone glue - Results

Impact on the adhesive strength of the bone glue

Sterilization is a critical step in medical device developing and manufacturing before clinical utilization, while effects of sterilization on the safety and efficacy of medical products are often ignored. EtO is very common sterilization method for moisture-sensitive materials such as our bone glue. Nevertheless, this treatment could also alkylate the active groups of the materials or leave the residue on the materials and affect its mechanical or/and biological properties. γ irradiation is an alternative technique of sterilization, which uses high energy electromagnetic radiation to ionize molecular component of the bacteria and generate extremely unstable free radicals to kill microorganisms. Contrarily to EtO, there is no residue left in the material after sterilization.

As shown in Figure 1, after EtO treatment, adhesive strength of both glues were significantly inferior to that made with non-sterile powders: for TTCP/OPS (1.83 ± 0.56 MPa *versus* 2.6 ± 0.6 MPa, p=0.0281) and for TTCP/OPS-nPDA (2.06 ± 0.35 MPa *versus* 3.32 ± 0.68 MPa, p=0.0003). Regarding the γ irradiation treatment, the impact on adhesive strength of the glue (sterile one *versus* non-sterile one) showed a similar tendency as EtO method albeit without statistical significance, for TTCP/OPS (2.06 ± 0.57 MPa *versus* 2.6 ± 0.6 MPa, p=0.065) and for TTCP/OPS-nPDA (2.66 ± 0.84 MPa *versus* 3.2 ± 0.68 MPa, p=0.083).

In this study, we demonstrated the negative effect of EtO sterilization on the adhesive strength of the glue, while γ irradiation method has less impact. Furthermore, EtO has been described to leave residues within the pore of CPC [1]. Thus, it can be concluded that, the choice

of γ irradiation sterilization method, which is also the preferred method for sterilizing CPC [2], may affect less the final performance of the developed material.

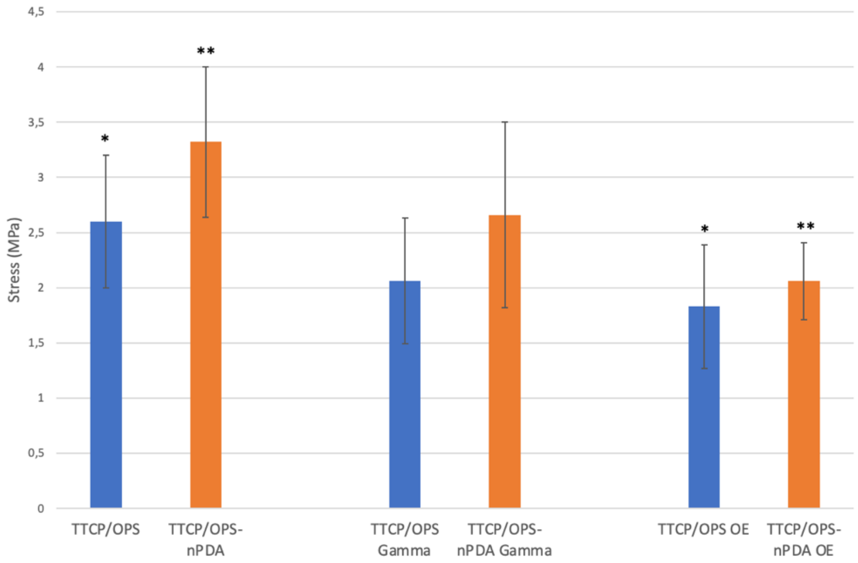

Figure S1: Instrumental traction test using titanium cylinders samples evaluating adhesive force of TTCP/OPS and TTCP/OPS-nPDA glue w/o treatment of γ radiation or ethylene oxide sterilization after 1-hour immersion in 37°C PBS. * or ** stands for statistically significant difference ($p=0.0281$ and $p=0.0003$ respectively) between two groups with the same symbol.

Sterility test

Microbiological assay showed that no bacterial growth was observed after 21-day of incubation in BHI broth for γ irradiated glue powders thus demonstrating that the bone glue fully met the requirement of sterility. On the contrary, for EtO treated powders, bacterial growth was detected from 7-day incubation on indicating that EtO method did not meet the requirement of sterility assurance level.

Therefore, taking all into consideration, γ irradiation method should be opted for sterilization of glue powder.

References:

[1] H. Takadama, M. Hashimoto, M. Mizuno, T. Kokubo, Round-robin test of SBF for in vitro measurement of apatite-forming ability of synthetic materials, Phosphorus Research Bulletin. 17 (2004) 119–125. https://doi.org/10.3363/prb1992.17.0_119.

[2] N. Eliaz, N. Metoki, Calcium Phosphate Bioceramics: A Review of Their History, Structure, Properties, Coating Technologies and Biomedical Applications, Materials (Basel). 10 (2017). https://doi.org/10.3390/ma10040334.
